# Zapping 500 faces in less than 100 seconds: Evidence for extremely fast and sustained continuous visual search

**DOI:** 10.1038/s41598-018-30245-8

**Published:** 2018-08-20

**Authors:** Jacob G. Martin, Charles E. Davis, Maximilian Riesenhuber, Simon J. Thorpe

**Affiliations:** 1 Centre de Recherche Cerveau & Cognition, CNRS-Université Toulouse 3, Toulouse, France; 2 Department of Neuroscience, Georgetown University Medical Center, Research Building, Room WP-12, 3970 Reservoir Rd. NW, Washington, District of Columbia 20007 USA

## Abstract

A number of studies have shown human subjects’ impressive ability to detect faces in individual images, with saccade reaction times starting as fast as 100 ms after stimulus onset. Here, we report evidence that humans can rapidly and *continuously* saccade towards single faces embedded in different scenes at rates approaching 6 faces/scenes each second (including blinks and eye movement times). These observations are impressive, given that humans usually make no more than 2 to 5 saccades per second when searching a single scene with eye movements. Surprisingly, attempts to hide the faces by blending them into a large background scene had little effect on targeting rates, saccade reaction times, or targeting accuracy. Upright faces were found more quickly and more accurately than inverted faces; both with and without a cluttered background scene, and over a large range of eccentricities (4°–16°). The fastest subject in our study made continuous saccades to 500 small 3° upright faces at 4° eccentricities in only 96 seconds. The maximum face targeting rate ever achieved by any subject during any sequence of 7 faces during Experiment 3 for the no scene and upright face condition was 6.5 faces targeted/second. Our data provide evidence that the human visual system includes an ultra-rapid and continuous object localization system for upright faces. Furthermore, these observations indicate that continuous paradigms such as the one we have used can push humans to make remarkably fast reaction times that impose strong constraints and challenges on models of how, where, and when visual processing occurs in the human brain.

## Introduction

It is often said that humans can typically make 2 to 5 saccades per second over a single scene^1,2^. During self-paced natural viewing conditions, humans make about 1–3 voluntary saccades each second^3,4^. Perhaps the best studied task for establishing saccade rates in humans are those involving reading. In the literature, there is evidence that highly skilled readers can average about 4.5 saccades each second when reading silently^5,6^. These continuous saccade rate estimates during reading included “saccadic regressions,” during which saccades occasionally moved in an opposite direction from the direction of reading (~11% of all saccades). Nevertheless, the direction of saccades during reading is very predictable (especially at the start of a line), and therefore the visual system would presumably use less resources making such saccades. Indeed, during visual search on a single image, humans make between 2–5 saccades each second^1,7^. Wu and Kowler found a maximum mean targeting rate of 3.3 circles a second when subjects tried to continuously make saccades towards oriented bars embedded in a portion of a group of small circles^8^. On the other hand, the number of *successfully* targeted circles with oriented bars per second in Wu and Kowler’s study reached a maximum mean of about 2.5 circles with oriented bars a second (see their Figure 1A,C).

However, are such numbers strict limits on the oculomotor system, or could the saccade rate be even higher with particular visual targets? Several studies have already shown speed and accuracy preferences for faces over other classes of visual targets. In a saccadic choice task, horizontal eye movements towards images containing face targets were particularly fast, and there were significantly more correct than incorrect saccades to faces as early as 100 ms after the onset of two images (which were not particularly small at 14° × 14° of visual angle) that were shown for 400 ms to the left and right of a fixation cross^9^. The same task also indicated a hierarchy of both saccadic reaction times (147 ms for face targets, 170 ms for animal targets, and 188 ms for vehicle targets) and saccadic accuracies (95% for face targets, 82% for animal targets, and 75% for vehicle targets). Thus, faces provoked faster and more accurate saccades compared to other visual objects. Yet, these saccadic choice paradigms typically use long intervals of pauses during which the subject is not doing the task of interest: a period of 1000 ms between trials, 800–1600 ms of a fixation cross, and a 200 ms “gap” with a blank screen between the fixation cross and the stimulus onset^9,10^. The duration of the blank screen, or “gap,” between a pre-trial fixation period and the onset of the visual stimuli can influence the resulting saccadic reaction times, with a 200 ms gap commonly showing the fastest reaction times^3,11^. Therefore, given the fast detection speed for individual faces, we used a continuous face detection task to better establish an upper limit for how fast the visual system can continuously perform visual search for complex objects.

While many studies have focused on the surprising speed and accuracy of object detection and recognition^9,12^, none to our knowledge have investigated the number of correct saccades towards objects embedded in changing scenes that can be made each second *continuously and without significant experimental pauses between trials*. In contrast to the previously mentioned paradigms, in which participants had to rapidly detect visual targets within an isolated image on each trial^9^, the behavioral paradigms in the current study did not have a 200 ms “gap” before the next trial, there were no significant pauses between trials, there was no previous training, the target locations and background scenes changed on every trial, and the subjects had no foreknowledge of the position of the target in each trial. Using a gaze-contingent paradigm with a high definition eye-tracker, we presented detection and localization tasks to the subject as rapidly as they could solve them. The next detection task was presented based on the previous target’s location and was presented as soon as the computer could detect that the subject’s eye reached the target location. Thus, our experiment sought to determine whether the visual system works as fast *continuously* as has been observed in paced experiments, and whether the continuous detection rate is faster for particular types of targets (i.e., upright faces versus inverted faces).

Our study measured the rates at which humans can continuously saccade towards sequentially shown faces in changing scenes. We investigated this new paradigm of continuous saccadic search in the context of either pasting and/or blending the faces into different background scenes, or pasting them on a simple gray background. Secondly, we used inverted faces as controls to test for advantages in continuous search for upright faces in comparison to inverted faces^13–16^, as these effects have only been shown previously in non-continuous paradigms. Furthermore, the inverted faces served as controls that allowed us to compare our results with the predictions of saliency models of human attention and saccadic choice^17^ because these models generally predict similar targeting preferences and performance for inverted versus upright faces in the context of no background scenes. We found that humans could continuously perform face detection and localization at surprisingly fast and sustained rates by making eye movements (up to 5.4 face targets/second when we hid the faces in a background scene, and up to 6 face targets/second when there was no background scene). The maximum face targeting rate ever achieved by any subject during any sequence of 7 faces during Experiment 3 for the no scene and upright face condition was 6.5 faces targeted/second. During this “ultrafast targeting period” of 6.5 faces targeted/second, there was only an astonishingly small amount of time, 154 ms, for the processing, localization, and the targeting of each face. The results confirm that there are performance differences between upright and inverted faces in the continuous setting and provide timing constraints on the neural circuitry underlying continuous visual search.

## Results

**Figure 1:**
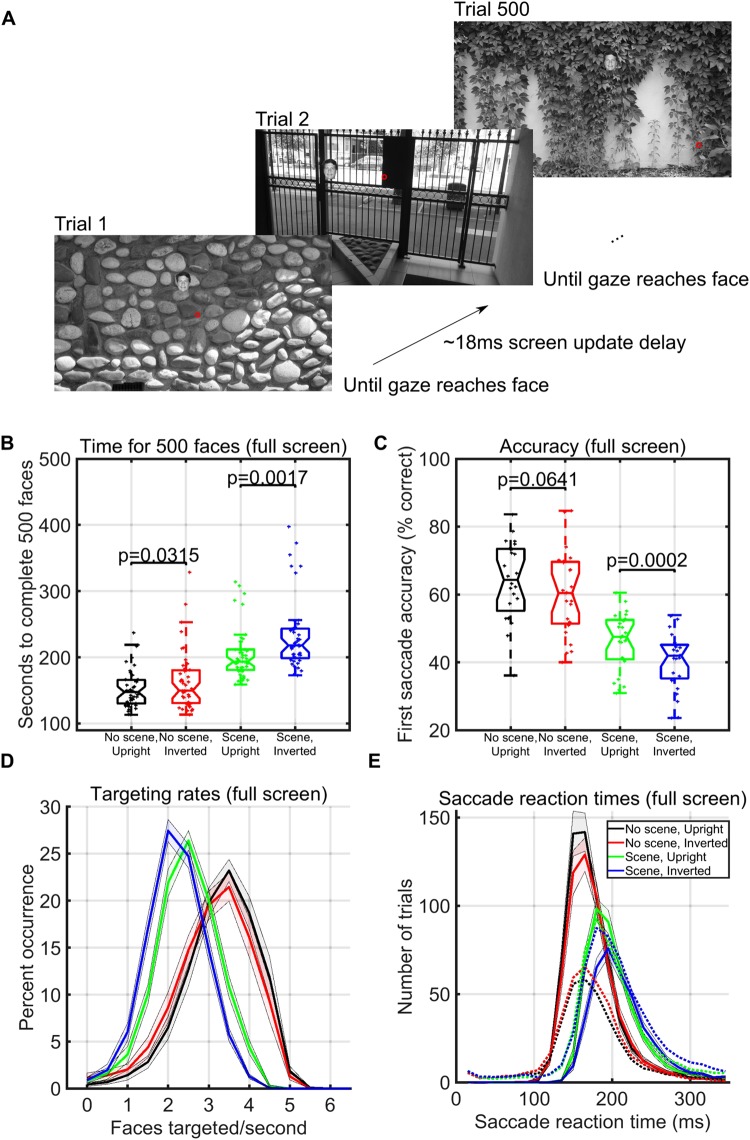
Paradigm and timing results over the entire screen (Experiment 1). (**A**) One block of 500 trials of the gaze-contingent continuous saccade detection paradigm with upright faces pasted on varying background scenes. The red circle represents the eye gaze location and did not appear during the experiment. The subject had to target the face with their gaze to proceed to the next trial. Locations of the 3° faces on each trial were independent from the previous trial and appeared at any location on the screen. Faces shown are for illustration, but all experiments used different faces in each trial of each block. (**B**) Boxplots of the number of seconds required, including blinks and all eye-movement times, to complete 500 3° faces in each condition over 24 subjects in Experiment 1. Individual dots are the time taken to complete a single counterbalanced block of 500 faces in each particular condition for each subject. (**C**) Percentage of trials in which the first saccade after stimulus onset landed within a 3° × 3° square surrounding the face (see Methods), for each condition. Shaded areas represent the standard error of the mean over the 24 subjects. (**D**) Percent occurrence of achieving a given rate of face detection speed (including all successes, mistakes, blinks, and eye movements) according to each condition (calculated over all possible sequences of 6 faces for each of the 24 subjects in the experiment). (**E**) Mean subject-specific saccade reaction time trial counts within all possible 10 ms wide time windows between 0 and 350 ms for correct saccades which landed within a 3° × 3° square surrounding the face (solid lines) and those that did not (dotted lines), separated by experimental condition. Shaded areas represent the standard error of the mean over the 24 subjects.

We ran three separate experiments designed to explore the speed of continuous saccadic face detection (N_1_ = 24 subjects, N_2_ = 24 subjects, N_3_ = 24 subjects). In all experiments, subjects continuously localized 500 different inverted or upright faces in counterbalanced blocks (Fig. 1A). There were 8 blocks, and each block had 500 different upright or inverted faces which were either *directly pasted* into one of 500 different cluttered background images or were pasted only on a gray screen. As soon as the subject’s gaze arrived at the face on the screen, the next trial was shown in about 18 ms (Fig. 1A). More specifically, to move on to the next trial during the experiment, the stimulus computer had to continuously communicate with the eye tracker computer to detect whether the subject’s gaze was located within a square of size 3° × 3° centered on the face, and then update the screen with the next trial. During analysis after the experiment, we considered that the first saccade after stimulus onset was correct if and only if it landed within the 3° × 3° square centered on the face.

**Figure 2:**
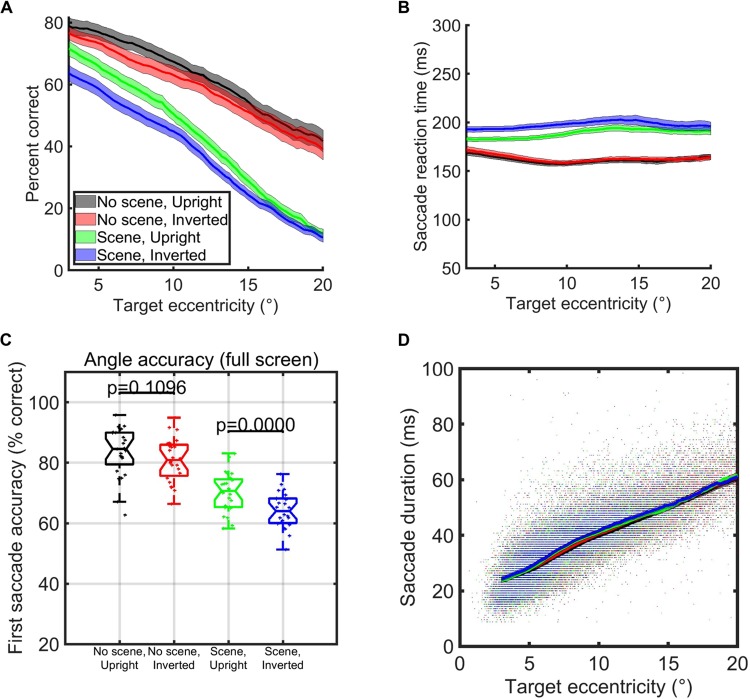
Eccentricity and saccade timing analyses over the entire screen (Experiment 1). (**A**) Percent of trials that the first saccade after stimulus onset landed on the face at each eccentricity, colored according to experimental condition (see legend). Error bars for each condition correspond to the standard error of the mean of the subject averages when targets were within 2° windows surrounding each point on the x-axis. (**B**) Mean saccade reaction time according to each eccentricity of the stimulus. Error bars correspond to standard error of the mean of the subject averages within 2° windows for that condition surrounding each point on the x-axis. Red and black curves overlap to some extent. (**C**) Angle accuracy of the first saccade after stimulus onset. Here, we counted the first saccade after stimulus onset as accurate whenever its angle was within π/8 radians of the angle of the face with respect to the eye position at the start of the trial. (**D**) Scatter plot for correct trials comparing target eccentricities and saccade durations according to condition (colors – see legend). Shaded areas are the standard errors of the subject means of the y-axis (row variable) over all correct trials within a window of two standard deviations around each particular point on the x-axis (column variable).

In Experiment 1, each trial contained a single 3° face pasted at any possible location on the screen, covering eccentricities from 4° to 16° from the previous fixation. To complete an entire block of 500 faces, subjects took an average time of 152 seconds (upright faces pasted on gray backgrounds), 164 seconds (inverted faces pasted on gray backgrounds), 204 seconds (upright faces pasted on background scenes), and 233 seconds (inverted faces pasted on background scenes, see Fig. 1B). Despite the large range of eccentricities used, subjects targeted up to 4.8 upright faces each second when we pasted the faces on background scenes and up to 5.5 upright faces each second when we pasted the faces on a gray background scene (Fig. 1D). The distributions of average face targeting speeds all passed tests for normality (Anderson-Darling Tests), and all paired t-tests of subject averaged rates of the number of faces targeted each second between conditions were significantly different (p < 0.02, df = 25). In the context of a background scene, upright faces were detected the fastest, with face inversion effects in average saccade reaction times, average targeting rates, average block completion times, and average accuracies from 4° to 16° eccentricities (Figs 1 and 2A). In addition, when we pasted the faces on a simple gray background, there were significant differences between upright and inverted face conditions in average completion times (t-test, p = 0.03, df = 25) and targeting rates (t-test, p = 0.02, df = 25); but only a trend towards a significance difference in the accuracy of the first saccade (t-test, p = 0.06, df = 25) (Fig. 1B,C,D).

Figure 2A shows the accuracies of the first saccade by eccentricity and condition (defined as whether or not the first saccade landed within a 3° × 3° square surrounding the face). Saccade accuracy based on the *angle* of the first saccade was higher than the more stringent requirement that saccades land on the face (Fig. 2C). In terms of saccadic undershoot^18^, we found the typical effect wherein undershoot magnitude positively correlated with the target eccentricity (see Figure S1C). The amount of undershoot was also significantly different according to whether or not there was a background scene at larger eccentricities (Figure S1C). From the histograms shown in Figure S1B, it is clear that most saccades that started with the correct polar angle (defined as those saccades which were within π/8 radians around the true polar angle) landed on their target, but that there were more undershoots than overshoots. We also plotted the average number of undershoots, overshoots, and correct saccade endpoints for saccades which were in the correct direction (as defined by π/8 radians – Figure S1A). Clearly, most saccades that started in the correct direction landed on the face area, although there were also many undershoots. For corrective saccades, we analyzed the first saccade that had the correct polar angle towards the face target. Focusing on initial undershooting saccades (which had the correct angle); we calculated accuracy for the following corrective saccade as whether the corrective saccade landed in the face target area. The results for these corrective saccades that occurred after undershooting (or overshooting) appear in Figure S1D (respectively, Figure S1E).

When grouped by target eccentricity, we found the expected negative correlation of accuracy with target eccentricity (Fig. 2A). As expected, saccade duration, defined as the time from saccade start to saccade end, increased with eccentricity (Fig. 2D; Pearson correlation coefficients for upright faces on background scenes: r = 0.76237 p = 0, inverted faces on background scenes: r = 0.75349 p = 0, upright faces on gray backgrounds: r = 0.5672 p = 0, inverted faces on gray backgrounds: r = 0.51543 p = 0). Interestingly, reaction times for correct saccades increased by only a small amount with eccentricity when the faces were pasted into a background scene (Fig. 2B; Pearson correlation coefficients for upright faces: r = 0.08, p = 1.325e-34, inverted faces: r = 0.04, p = 7.7876e-11). When there was no background scene, saccade reaction times were even a bit faster as eccentricities increased (Pearson correlation coefficients for upright: r = −0.05, p = 6.8388e-15, inverted: r = −0.07, p = 1.6833e-28).

**Figure 3:**
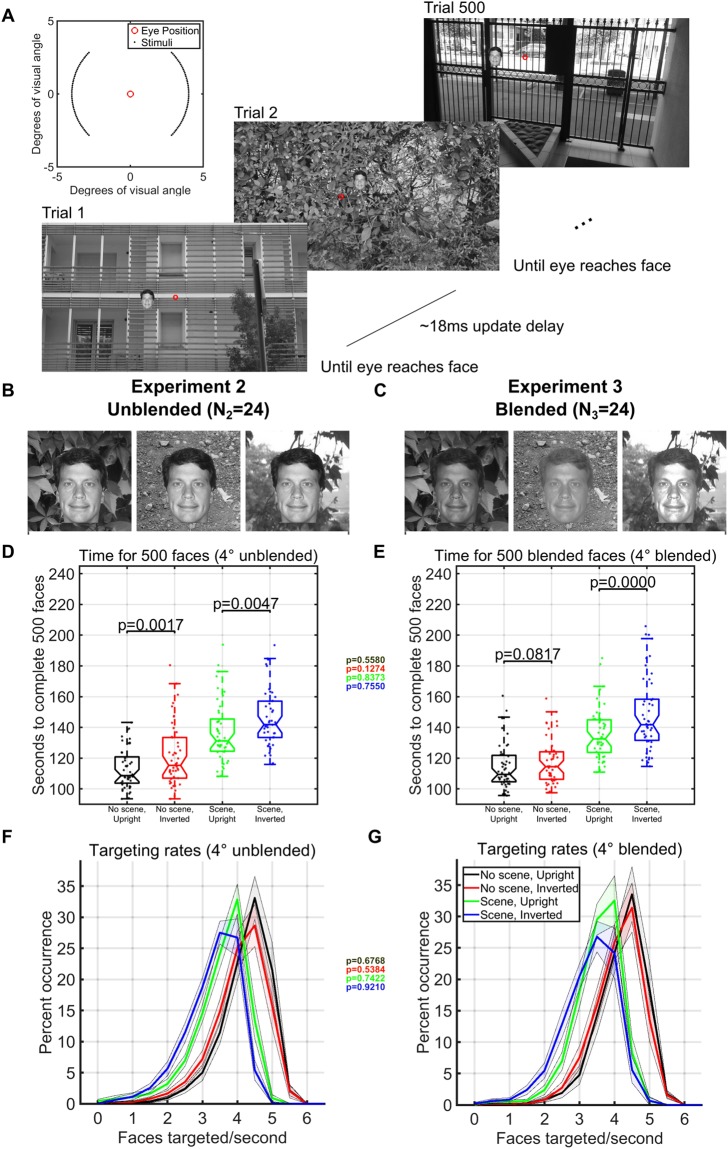
Paradigm and timing results in limited eccentricities and polar angles (Experiments 2 and 3). (**A**) One block of 500 trials in the gaze-contingent continuous face detection paradigm with upright faces pasted on a background. The red circle represents the hypothetical gaze location and did not appear during the experiment. The subject had to localize the face with their gaze to move on to the next trial. Locations of the 3° faces were within 4° eccentricity of the location of the face in the previous trial and appeared at polar angles between 0°–45°, 135°–235°, 315°–360° (see inset at upper left). Faces shown are for illustration, but all experiments used different faces in each trial of each block. (**B**) Examples of how unblended faces would appear on different backgrounds in Experiment 2 (N_2_ = 24 subjects). (**C**) Examples of how blended faces would appear on different backgrounds in Experiment 3 (N_3_ = 24 subjects). (**D**,**E**) Boxplots of the number of seconds required, including blinks and all eye-movement times, to complete 500 3° faces at 4° eccentricities in each condition over 24 subjects in Experiment 2 (**D**) and Experiment 3 (**E**). Individual dots are the time taken to complete a single counterbalanced block of 500 faces in each particular condition for each subject. Text inset of colored p-values shows the statistical comparisons for each color-coded condition between the mean single session completion time for Experiment 2 (unblended) and the mean single session completion time for Experiment 3 (blended) using only the 17 subjects that only did one experiment (separate Wilcoxon ranksum tests for each condition). (**F**,**G**) Probability of achieving a given rate of face detection speed (including all successes and mistakes) according to each condition calculated over all possible sequences of 6 faces for each of the 24 subjects in each experiment (left histogram plot for unblended faces, N_2_ = 24, right histogram plot for blended faces, N_3_ = 24). Text inset of colored p-values shows the statistical comparisons for each color-coded condition between mean face targeting rates for Experiment 2 (unblended) and mean face targeting rates for Experiment 3 (blended) using only the 17 subjects that only did one experiment (separate Wilcoxon ranksum tests for each condition).

**Figure 4:**
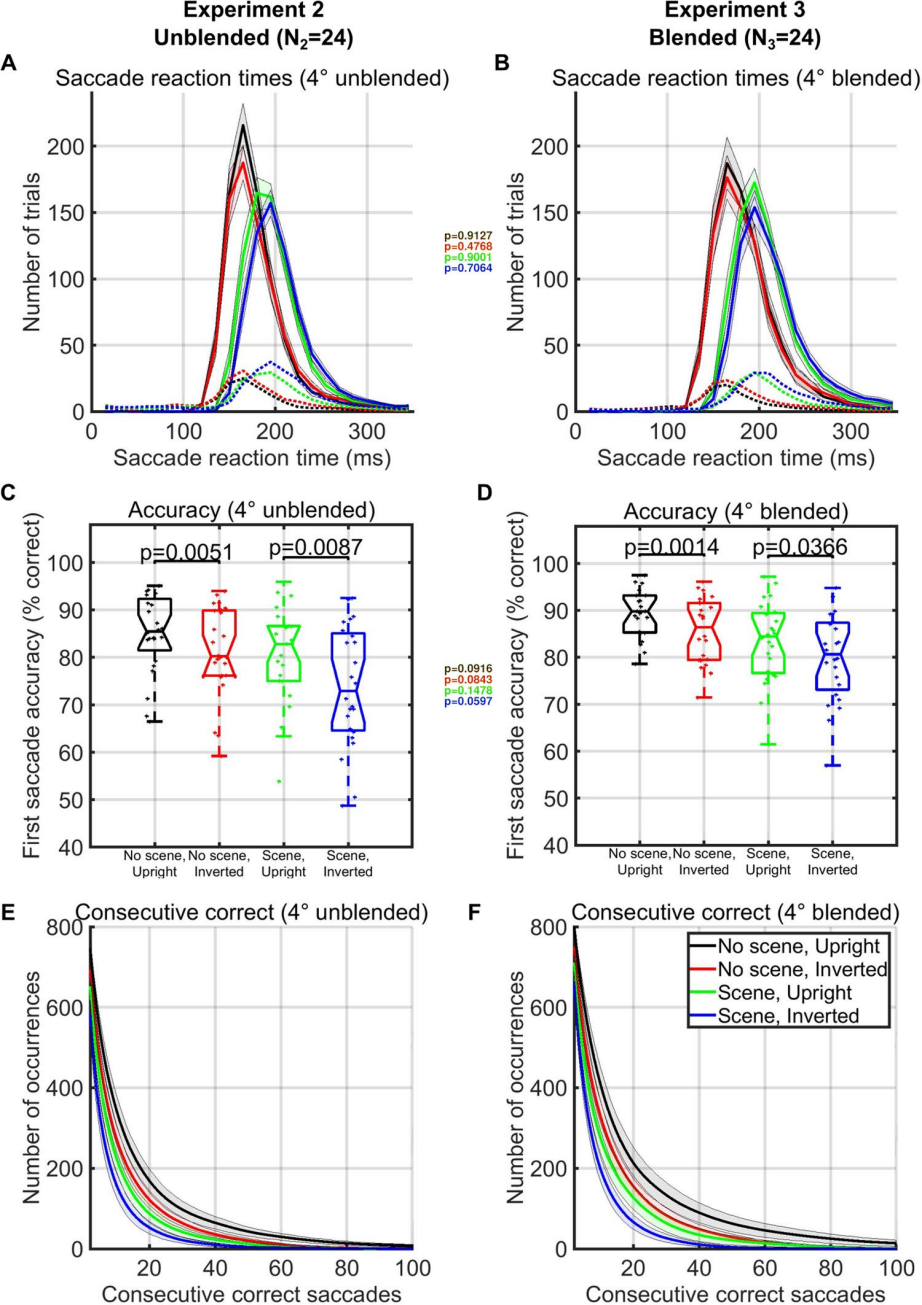
Saccade reaction time and first saccade accuracy comparisons for Experiment 2 and 3 (limited eccentricities and polar angles–see Fig. 3A, inset). (**A**,**B**) Saccade reaction time histograms for all trials and all subjects ((**A)**: unblended, Experiment 2, (**B**): blended, Experiment 3). Solid lines represent correct trials and dotted lines represent incorrect trials. Each color represents a different stimulus condition (see legend). Text inset with colored p-values shows the statistical comparisons for each color-coded condition between mean subject SRTs for Experiment 2 and mean subject SRTs for Experiment 3 using only the 17 subjects that only did one experiment (Wilcoxon ranksum test). (**C**,**D**) Accuracy of the first saccade for each condition as defined by whether or not the first saccade after stimulus onset landed within a 3° × 3° square surrounding the face. Text inset of colored p-values shows the statistical comparisons for each color-coded condition between Experiment 2 and Experiment 3 using only the 17 subjects that only did one experiment (Wilcoxon ranksum test). (**E**,**F**) Number of occurrences of each particular length of consecutive correct saccades for each condition (x-axis). Shaded areas represent the standard error of the subject mean at each location for each condition.

The rates of continuous face detection in Experiment 1 included trials in which large eye movements had to cross the entire screen (see Fig. 2D). Therefore, we did another study (Experiment 2) that we designed to increase the number of faces targeted each second by presenting each subsequent face at a fixed eccentricity of 4° from the previous face (see Fig. 3A). Also, the polar angles were set such that subsequent targets always appeared at polar angles of 0°–45°, 135°–235°, 315°–360° from the previous target (Fig. 3A, inset). These experimental manipulations increased the number of faces that participants targeted each second when there was a background scene from a maximum of 4.8 faces targeted/second over the entire screen (Experiment 1, Fig. 1D), to a maximum of 5.4 faces targeted/second (Experiment 2, Fig. 3F). Targeting rates approached 6 faces a second when faces were presented upright and without a background scene, and were faster for upright faces versus inverted faces in both background scene (t-test, p = 0.0007, df = 25, Fig. 3F) and no-background scene conditions (t-test, p = 0.0005, df = 25, Fig. 3F). On average, subjects took 111 seconds to localize 500 3° upright faces with no background scene (N_2_ = 24, Fig. 3D), increasing to 137 seconds when the upright faces were pasted on cluttered background images. Additionally, the number of correct *consecutive* saccades for upright faces was almost double that of inverted faces, whether or not there was a background present (Fig. 4E).

To control for potential pop-out effects, we conducted a 3^rd^ experiment in which we further hid the faces in the scene by matching their grayscale histograms with those of the local histogram in the background image in which it was pasted (Fig. 3C). As in Experiment 2, we placed each face at an eccentricity of 4° from the previous face. In the third experiment, the fastest subject processed a block of 500 upright faces in only 96 seconds with no background scene and in only 111 seconds when we blended the upright faces into the local background scene (Fig. 3D,E). Despite the faces being further hidden with blending, targeting rates remained significantly different between upright and inverted faces in both the background scene condition (t-test, p = 0.0004, df = 25) and no-background scene condition (t-test, p = 0.028, df = 25). Remarkably, a comparison between the 17 subjects that only participated in one of the 2^nd^ and 3^rd^ experiments indicated that blending the faces into the local background had no significant effects, according to Wilcoxon ranksum tests, between saccade reaction time (p > 0.48), targeting rates (p > 0.54), completion times (p > 0.13), or first saccade accuracies (p > 0.06, Figs 3 and 4–insets).

## Discussion

In this paper, we have described results from a new psychophysical paradigm that shows that participants can continuously detect and saccade to faces at rates up to 6 faces/second with no background and up to 5.4 faces/second when we hid or blended the faces into a background scene. The title also introduces a new name, “Zapping,” for the paradigm to reflect the fact that once the face was targeted, the face was nearly immediately removed (“zapped”), and a new face, in a new and unpredictable location, was immediately presented. Subjects described the experience as being on “autopilot,” “effortless,” or “like they were a machine.” In this new behavioral paradigm, participants completed an enormous amount of trials in a very short amount of time. For example, our study encompassed 288,000 trials done by 72 subjects, and yet only took approximately 40 hours of recording.

The zapping paradigm used in this paper is not directly comparable to the results reported from previous paradigms of visual search, but it is still instructive to make comparisons with previous results which report the number of items that can be visually targeted each second. Most visual search studies have used static sets of visual objects containing both distractors and targets which appeared together on a fixed screen which did not change when the target(s) were found. These paradigms have reported search rates that range from 3–4 items per second^7^. One study from Wu and Kowler reported that the average rate of the number of circles targeted per second was 3.3 circles/second (see Fig. 4b from their paper)^8^. However, that study used fixed positions for the circles, there was no change in the circle locations as circles were fixated, subjects only performed the task for 1 or 2 seconds, and all trials containing blinks were removed (and thus their results were not affected strongly by blinks or fatigue). Moreover, when the search rate was calculated according to the number of targets (oriented bars within a certain portion of the circles) found per second, the average number of targets per second found was 2.25 oriented bars/second (see Fig. 4c of their paper)^8^. There are several important additional distinctions between the current study’s experiments and these previous experiments. First, the measure that we used in 3 F and 3 G was “faces target/second;” which included all saccades, blinks, microsaccades, saccade duration, screen update times, and corrective saccades (as opposed to other studies which excluded trials with blinks or excluded “infrequent instances of revisits to a location,” e.g.^8^). Second, we calculated targeting rates within continuous periods of around 100–120 seconds for each block (as opposed to within 1 or 2 second periods after long (e.g. 400 ms) breaks in between trials). Third, the positions of the face targets in our task were not predictable (as opposed to fixed locations which did not change between trials). Fourth, the zapping paradigm’s single target detection tasks with changing background scenes is arguably harder than the previously mentioned tasks because the targets of interest were hidden or blended into cluttered background scenes.

Despite the fact that the zapping task is arguably harder than these previous studies, the average face targeting rate that we observed in Experiment 3 was 4.4 faces targeted/second (33% higher than the reported 3.3 circles targeted/second and 95% higher than the reported 2.25 oriented bars targeted/second). When we made the task harder by using a background scene, we found 3.7 faces/second (12% higher than the reported 3.3 circles targeted/second and 64% higher than the reported 2.25 oriented bars targeted/second). For the blended condition in Experiment 3, there were 414 times when subjects targeted faces at speeds greater than 5.5 faces a second. The maximum rate ever achieved by any subject during any sequence of 7 faces during Experiment 3 for the no scene and upright face condition was 6.5 faces targeted/second (97% higher than the reported 3.3 circles targeted/second and 189% higher than the reported 2.25 oriented bars targeted/second). If we consider the upper bound reported in the literature as 4 or 5 items targeted each second (see, e.g., reference^7^), although other studies have reported less, then it seems clear that even though our task is arguably harder (no breaks, continuous saccades, changing screens, wide varieties of target locations, blink durations, *etc*.), we still obtain a new bound of 30% above the highest reported rate in the literature (6.5 versus 5.0). Furthermore, we believe that it is quite likely that both technological (eyetracker, gsync, faster displays, *etc*.) and experimental modifications (eccentricities, sizes, stimuli categories) can be made to this paradigm to achieve even faster visual search and targeting speeds in the various conditions (background, no background, blended, *etc*.), and that our results are only a new candidate upper bound for the fastest targeting rates that may truly be possible.

It is possible that this difference in targeting rates is driven by the fact that we used faces as stimuli, as faces have been shown to provoke faster saccade reaction times than other stimuli such as animals or vehicles^9^. Nevertheless, the results show that humans can continuously target faces almost as fast as has been found in slower-paced non-continuous experiments. A rate of 5.4 faces a second corresponds to 185 ms per target, including eye movement times (~30 ms at 4*°* eccentricity, see Fig. 2D) and screen update delays (~18 ms), leaving ~137 ms of processing time for each of the targets. Surprisingly, hiding the faces further by matching their histograms with that of the local histogram around the pasted location did not significantly affect the overall speed, accuracy, targeting rates, or saccade reaction times of subjects in the two experiments which used 4 degree eccentricities.

Traditionally, paradigms investigating saccade speed have utilized relatively large inter-trial intervals (1–2 seconds) and various other mechanisms of inter-trial blanks and fixation periods that left open what sustained continuous object detection rate the human visual system could achieve. Although inserting a “gap” of 200 ms after the fixation and before the stimulus has been found to decrease saccade reaction times^10,11,19^, these pauses slow down the experiment and force subjects to allocate cortical resources to maintaining fixation. Yet, even without the addition of the 200 ms blank screen before the stimulus onset, continuous face detection in our experiment was fairly close to the speeds reported for paced detection in previous experiments. We also found that participants localized upright faces faster and more accurately than inverted faces across a wide range of eccentricities and contexts (blended/non-blended, background scene/no background scene). While humans have shown behavioral advantages for upright faces over inverted faces in non-continuous settings^13,14^, our results extend this advantage to a continuous setting. In the first experiment, we verified that the advantages for the targeting of upright faces compared to inverted faces also occurred across a large range of eccentricities (Figs 1 and 2). These advantages for upright faces were apparent when comparing the number of correct consecutive saccades made in a row across conditions (Fig. 4E,F) and were present whether stimuli were pasted all over the screen (Experiment 1) or pasted at a fixed 4-degree eccentricity from the previous face (Experiments 2 and 3). The existence of such a difference between two closely matched and controlled visual objects point towards the idea that face-specific neural processes implement upright face detection, and perhaps is not solely reliant on a general-purpose machinery or standard neural hierarchy for general multi-class object detection. Given that detection rates for faces were so fast in this continuous setting and that there were clear advantages for processing upright faces, one possibility is that the early visual cortex contains optimized pathways, maybe even cortical “shortcuts”, in the form of neural circuits or special purpose neurons, which code for the most frequently-experienced or ecologically relevant stimuli.

How might one juxtapose our results with the various existing models of saccadic execution during visual search? The saliency model from Itti and Koch uses low-level visual features like intensities and orientations of a single image to predict locations of covert attentional focus and human eye movements^17,20^. This bottom-up model has inspired several associated models of attention and saccadic processing^21–23^. However, low-level saliency models do not explain the speed and accuracy differences we found between upright and inverted faces presented on gray backgrounds, because the low-level salience maps of these two conditions are identical. In addition, the observed absence of an effect of histogram blending on face detection rate and performance found between Experiments 2 and 3 appears difficult to reconcile with a low-level saliency account that would predict reduced saliency for faces when they are histogram-matched to their surrounding background. On the other hand, Cerf *et al*. reported that a low-level saliency model *combined* with a face detection model was superior to a low-level saliency model alone when predicting human fixations in scenes containing faces (e.g. 0.68 ROC area vs. 0.80 ROC area)^24,25^. How might the brain accomplish such rapid and continuous face detection? Already, in 2001, Delorme *et al*. reported the feasibility of a simple and shallow spike-based model that could quickly process faces while gracefully handling image degradations by showing robustness to noise and changes in contrast^26,27^. Such a simple spike based model can also account for the rapid behavioral speeds we observed in our study. Although the idea that face detection happens early in the visual hierarchy is not a common one, some evidence that this may be the case arises from the extremely fast 100 ms saccade speeds to faces found in Crouzet *et al*.^9^, the ability to detect face location starting at only 50 ms after stimulus onset in EEG signals^28^, and the fast continuous face targeting speeds and high localization accuracies found in our study. These results also support the intriguing hypothesis that there may be face-selective neural representations in early visual areas.

Why is it important to study the speed of the visual system, and why does it matter that we have found faster targeting speeds than have been previously reported? It is our view that the task we used in this paper is one of the fastest behavioral tasks that human subjects can possibly do. Most strikingly, the continuous face-targeting rates we found in our experiments were faster than the 2–5 targets a second reported in other studies^2,8^. These ultra-rapid targeting rates and the behavioral differences between upright and inverted faces occurred despite the matching of low-level saliencies. Therefore, our results impose strong constraints on how, when, and where the brain accomplishes visual object detection; and further challenge and provide impetus to improve existing oculomotor models of visual saccadic search.

## Materials and Methods

We conducted three separate experiments designed to explore the speed of continuous face detection (N_1_ = 24 subjects, N_2_ = 24 subjects, N_3_ = 24 subjects). In all experiments, subjects continuously localized 500 inverted or upright faces in blocks where the faces were either *directly pasted* into one of 500 different cluttered background scenes, or used only a gray screen as the background. The first experiment used a large range of eccentricities and polar angles, so that each face could appear anywhere on the screen and the face’s location was not based on the previous trial (see Figure 1A). In the second experiment, we pasted faces directly on the backgrounds, whereas in the third experiment we also locally *blended* the faces into the image by matching their grayscale histogram distribution to that of the local histogram at the pasted location (Fig  3B,C). To minimize the time for saccade movements, every subsequent face in the second and third experiments appeared only 4° in eccentricity away from the previous face. Also, the polar angles in the second and third experiments were set such that subsequent targets appeared at polar angles of 0°–45°, 135°–235°, 315°–360° from the previous target (Fig. 3A, inset).

### Stimuli

We created the face stimuli from a set of 2316 images of segmented faces from the Humanae project (with written permission from the artist Angélica Dass, http://humanae.tumblr.com/)^29^. Background image stimuli for all experiments were selected from a large database of images containing 861 images, some of which have been used in previous psychophysical studies^30^. We converted the faces and backgrounds to grayscale. We resized faces to have a height of 3*°* of visual angle. We resized image backgrounds to cover the entire screen resolution of 2560 × 1440 pixels.

### Presentation hardware

Stimuli were presented on an ASUS ROG Swift PG278Q GSYNC monitor with 1 ms response time and 120 Hz refresh rate, driven by two SLI linked NVIDIA GeForce 980GT GPUs, at a screen resolution of 2560 × 1440^31^. The display subtended approximately 31 degrees horizontal and 22 vertical degrees of visual angle. The display was controlled by a custom-built workstation running Gentoo Linux with a 64 bit kernel that was tuned for real-time processing. The paradigm was programmed in Matlab R2008a (The Mathworks, MA) using Psychtoolbox version 3^32,33^. We recorded target onset presentation times with a photodiode that was time-synchronized with the eyetracker.

### Eyetracking

We recorded eye movements using the SMI iViewX High Speed system with a 1250 Hz sampling rate on the subject’s dominant eye. Before the first session, we determined each subject’s dominant eye and subsequently recorded and calibrated only that eye. The eye-tracker recorded the dominant eye at a rate of 1250 Hz and sent gaze position samples at a delay of approximately 5 ms to the presentation hardware. We compared the time of the entrance of the eye within the target face area and the subsequent photodiode onset for the next trial to identify that the median screen update time was 18.03 ms. These values did not differ by more than 1 ms when examined by condition (e.g. for Experiment 1: 17.67 s, 17.95 ms, 18.09 ms, and 18.42 ms).

Saccades were detected only after the experiment. During the experiments, only the gaze position data were used to advance to the next trial. After the experiment, saccades were detected using the procedure and parameters described below. Furthermore, all saccades detected after the experiment with amplitude less than 1 degree were considered microsaccades and were removed from analyses of saccade reaction time and saccade accuracy. All accuracy numbers that we reported were based on the first saccade (not microsaccade) after stimulus onset. In Experiments 2 and 3, we wanted to make the paradigm and subjects go as fast as possible by minimizing saccade durations and making the faces appear closer to the fovea than they did in Experiment 1, so we chose 4 degree eccentricities and 3 degree faces to task the subjects with making a “saccade,” which likely would not be a microsaccade, to go on to the next trial. Note however, that the position of the next face after a border-targeting saccade could be either larger or smaller in eccentricity with respect to the gaze location because the position of the next face was random. However, an “actual eccentricity analysis,” which computed eccentricity according to the gaze position at stimulus onset, indicated that stimuli were almost never presented in the microsaccadic range. There were very few trials in which the center of the next face was less than 2.51 degrees eccentricity from the eye position at stimulus onset (around 0.25% of all trials). Because subjects had to saccade within the center of a 3 × 3 degree square surrounding the next trial’s face, very few of the eye movements extracted from the experiment, or during analyses, could have been simultaneously a microsaccade and also considered correct during the experiment. There were only 0.01% (i.e. 89/96000) trials in which there were microsaccades away from the target during the first 200 ms after stimulus onset in Experiment 3. Of the subsequent saccades after these microsaccades, 84.27% landed on the target. However, of all saccades detected, 84.37% of them landed on target. Thus, subjects rarely used a microsaccadic search strategy during the paradigm, and if they did, the strategy did not yield any improvement in accuracy over a single directed saccade. An exploration in terms of the time required for such a microsaccadic search strategy also proved illuminating. A recent paper from Gao *et al*. showed that microsaccade rates on non-continuous tasks (i.e., tasks that have pauses in between trials) drop dramatically (e.g., to less than 0.01 microsaccades/second) during the first 200 ms after stimulus onset and are modulated according to task difficulty^34^. Average overall rates found in such noncontinuous tasks are typically between 1–2 microsaccades each second. On the other hand, we found that subjects can target up to 5–6 faces each second–rates that are 3–6 times faster than the microsaccade rates published in the literature. Therefore, it does not seem likely that the subjects could have used a microsaccadic strategy during our study and yet achieved such high rates of search. To be certain, we counted the number of microsaccades that occurred during the first 200 ms after stimulus onset. In Experiment 3, 97.96% of trials did not contain a microsaccade in the first 200 ms after stimulus onset, 1.98% of the trials had one microsaccade, 0.05% of the trials had two microsaccades, and 0.01% of the trials had three or more microsaccades. Also, the average saccade amplitude after stimulus onset was 4.0031 degrees. Nevertheless, after the experiment, such microsaccades did not influence the calculations of saccade reaction times or saccade accuracies.

To detect saccades after the experiment we used the “microsacc plugin” for saccade detection with a smoothing level of 2 (“the raw data is smoothed over a 5-sample window to suppress noise”^35^), a velocity factor of λ = 5 to determine the velocity threshold for saccade detection (“thresholds were relative to the noise level, calculated as λ = 5 multiples of a median-based SD estimator”^35^), and a minimum saccade duration of 10 samples (corresponding to 8 milliseconds)^35^.

### Statistical design

Data points that were outliers (as detected by Rosner’s generalized ESD test) were removed before statistical testing^36^. For example, block times which were outliers (as detected by Rosner’s generalized (extreme Studentized deviate) ESD test) were removed to compute Fig. 3E (only 3 blocks across all conditions). Unless otherwise noted, nonparametric Wilcoxon rank-sum tests were used to compare subject averages across the different conditions for each covariate of interest^37^. We used the Matlab statistical toolbox (The Mathworks, MA) to implement each statistical test.

### Experimental design

A total of 44 subjects with normal or corrected-to-normal vision participated in a total of 72 separate sessions divided into 3 experiments: Experiment 1 (N_1_ = 24, two left-handed, 14 females, ages 21–39), Experiment 2 (N_2_ = 24, two left-handed, 10 female, ages 22–53), and Experiment 3 (N_3_ = 24, two left-handed, 13 female, ages 21–40). Some subjects participated in more than one of the three experiments: 6 took part in all three experiments, 9 in only Experiments 1 and 2, 6 in only Experiments 1 and 3, 1 in only Experiments 2 and 3, 3 in only Experiment 1, 8 in only Experiment 2, and 11 in only Experiment 3. Each experiment consisted of 4 different conditions (No Background, Upright; No Background, Inverted; Background, Upright; Background, Inverted) that were separated into separate blocks. Each block contained 500 trials all of a single condition. The orders of the blocks were counterbalanced across subjects so that each of the subjects did one of the possible 24 possible block orderings of the 4 conditions. This same order of 4 blocks was then repeated in another section of 4 blocks, so that subjects did a total of 8 blocks. After each block of 500 trials, there was a small pause of about 2 minutes while we recalibrated the eye tracker to ensure that the calibration remained accurate throughout the experiment.

Within each session, participants performed a continuous detection task in which we pasted the 3*°* tall face stimuli into large scenes that filled the entire 2560 × 1440 pixel screen of the monitor (31*°* horizontal and 22*°* vertical, see Fig. 1A). Each subsequent trial started immediately after the subject’s eye landed within the previous face (with a median screen-update error of 18 ms after the subject found the previous face). We randomly pasted the faces either based upon the position of the face in the previous trial (Experiments 2 and 3) or completely randomly within the 2560 × 1440 pixel scene (Experiment 1). All faces and background images within any given block were unique. We paired and combined the faces and background images before the block and counterbalanced them across subjects so that each face and background image combination appeared equally in every condition. Participants were told to find the faces with their eyes as fast as they possibly could.

To make the paradigm go as fast as possible, but also retain some robustness to noise, we required subjects to maintain gaze within the 3 × 3° region centered on the face for at least 2 samples (1.6 milliseconds). The eye tracker was sampling the gaze position at 1250hz. So, two consecutive gaze position samples had to remain in the surrounding face target area before the paradigm would move on to the next trial. Therefore, it is possible that a saccade sweeping across and out of the 3 × 3° face target area might lead to the presentation of the next stimulus. However, that saccade would not have been counted as correct during subsequent analyses of saccade accuracy (which depended on saccade velocity rate and saccade duration parameters in each trial to determine whether or not the first saccade after stimulus onset landed within the 3 × 3° region and was thus counted as correct). Therefore, the saccade reaction time and saccade accuracy results in the paper remain valid. On the other hand, an overshooting saccade, which passed through the target area surrounding the face and yet landed outside of the target area, would have been counted in the targeting rate calculations. However, as one can see from Figure S1B, there are very few trials in which this could have happened because most of the saccades that started in the correct direction undershot the target. To be certain of the potential effects on the targeting rate results, we counted the number of times that a saccade started in the correct polar angle, but overshot the target and was thus not counted as correct after the experiment. We found that this occurred in only 0.8% of all trials in Experiment 3. Figure 2D shows that saccade durations crossing a target window with a size of 3° to 4° should range from 10–40 ms, with an average duration of about 25 ms. Thus, a saccade crossing the target window with an area of at most 4.2426° (because the target area was 3 × 3° square) and at least 3° (i.e. from side to side) would provide ample time for the paradigm to move to the next trial. Therefore, within these 0.8% of trials in which the saccade overshot the target, the paradigm moved to the next trial during the experiment. However, this situation only occurred 0.8% of the time, which, if accounted for, is far less than the increases in rates we found versus other studies (please see the analyses in the paper where we find upto 189% increases over the published targeting rates of similar, but different, studies).

During the experiments, we did not place the faces based on the gaze position (which may have been better, but would have slowed the experiment down considerably given the considerable computational complexity required to blend and paste a face into a large 2560 × 1440 image at runtime). As subjects could have had their gaze anywhere in the 3 × 3 degree face detection window, the next trial’s face – which appeared at exactly 4 degrees eccentricity from the center of the previous face in Experiments 2 and 3–could have been presented further or closer to the actual eye position at stimulus onset. We have analyzed the distribution of actual eccentricities (from the subject’s current gaze location at stimulus onset). After the experiment, we were able to determine the actual eccentricity of the target according to the recorded gaze location at stimulus onset. All analyses of eccentricity used the gaze position at the time of stimulus onset. Despite the fact that we pasted images based on the previous target’s location, the average actual eccentricity indeed had a mean value of 4.12° during Experiment 2, and 4.09° during Experiment 3. We also checked that the actual average eccentricity did not differ by condition within each experiment (p > 0.11, Wilcoxon rank sum, df = 25).

The Committee for the Evaluation of Ethics of INSERM (CEEI Institutional Review Board) approved the experimental procedures and protocol, and we obtained written informed consent from all participants prior to each experiment. The experiments were performed in accordance with all relevant guidelines and regulations.

### Data availability statement

The datasets generated during and/or analyzed during the current study are available from the corresponding author on reasonable request.

## Electronic supplementary material

Supplementary Figure
