# Supplementary material for: Zapping 500 faces in less than 100 seconds: Evidence for extremely fast and sustained continuous visual search
Source: Sci Rep. 2018 Aug 20;8:12482. doi: 10.1038/s41598-018-30245-8 (PMC6102288; doi:10.1038/s41598-018-30245-8)
Supplement: Supplementary file 1 — Supplementary Figure [file 41598_2018_30245_MOESM1_ESM.docx]

Title: Zapping 500 faces in less than 100 seconds: Evidence for extremely fast and sustained continuous visual search

Authors: Jacob G. Martin^1,2,^*, Charles E. Davis^1^, Maximilian Riesenhuber^2^, Simon J. Thorpe^1^

**Affiliations :**

^1^Centre de Recherche Cerveau & Cognition, CNRS-Université Toulouse 3, Toulouse, France

^2^Department of Neuroscience, Georgetown University Medical Center, Research Building, Room WP-12, 3970 Reservoir Rd. NW, Washington, District of Columbia 20007, USA.

*Correspondence to: [jacobgmartin@gmail.com](mailto:jm733@georgetown.edu)

Funding by: NEI R01EY024161, ANR-13-NEUC-0004, and ERC Advanced Grant N^o^323711 (M4).


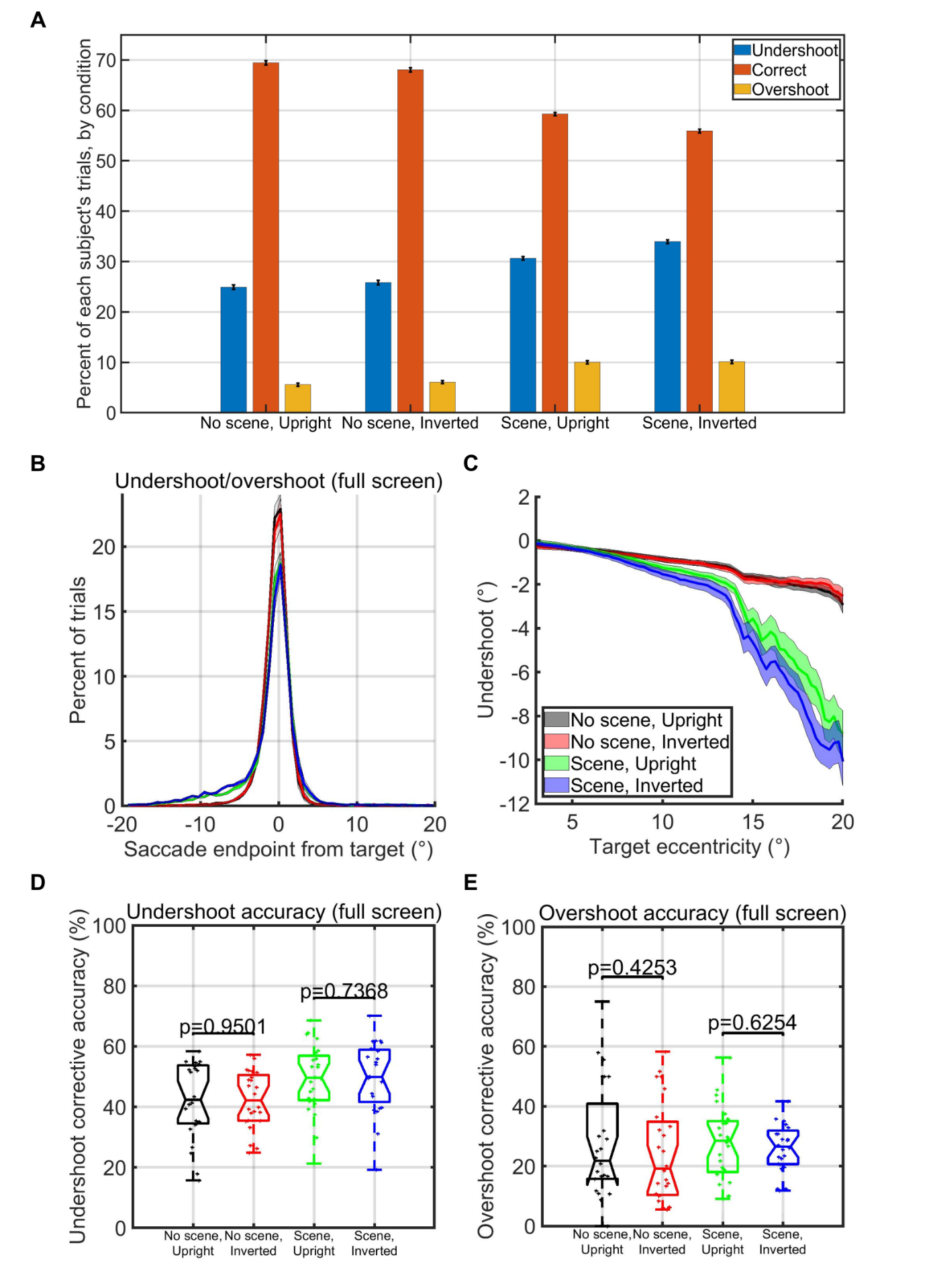


*Figure S1: Overshoot, undershoot and corrective saccade analyses for* ***Experiment 1.*** *(****A****) Mean percent of undershoot, on target (correct), and overshoot trials in each condition for each subject. These data are limited to the first saccades after stimulus onset that started in the correct angle (defined by those saccades that were less than π/8 radians from the true target location). Error bars represent the standard error of the mean. (****B****) Percent of trials (y-axis) having saccades that started in the correct angle and landed at each distance to the face target (x-axis). A negative degree target distance corresponds to undershoot and a positive degree target distance corresponds to overshoot. (****C****) Amount of undershoot/overshoot (y-axis) when averaging correct angled saccades according to 2 degree eccentricity windows (x-axis). (****D, E****) Mean subject accuracies of the corrective saccade given that the first saccade after stimulus onset was in the correct direction and yet undershot (overshot) the target. We considered a corrective saccade correct when it landed within a 3 x 3 degree square window surrounding the face.*
